# Proteomic Analysis of Skin Invasion by Blood Fluke Larvae

**DOI:** 10.1371/journal.pntd.0000262

**Published:** 2008-07-16

**Authors:** Elizabeth Hansell, Simon Braschi, Katalin F. Medzihradszky, Mohammed Sajid, Moumita Debnath, Jessica Ingram, K. C. Lim, James H. McKerrow

**Affiliations:** 1 Sandler Center, California Institute for Quantitative Biosciences (QB3), University of California, San Francisco, California, United States of America; 2 Mass Spectrometry Facility, Department of Pharmaceutical Chemistry, School of Pharmacy, University of California, San Francisco, California, United States of America; Queensland Institute of Medical Research, Australia

## Abstract

**Background:**

During invasion of human skin by schistosome blood fluke larvae (cercariae), a multicellular organism breaches the epidermis, basement membrane, and dermal barriers of skin. To better understand the pathobiology of this initial event in schistosome infection, a proteome analysis of human skin was carried out following invasion by cercariae of *Schistosoma mansoni*.

**Methodology and Results:**

Human skin samples were exposed to cercariae for one-half hour to two hours. Controls were exposed to water used to collect cercariae in an identical manner, and punctured to simulate cercarial tunnels. Fluid from both control and experimental samples was analyzed by LC/MS/MS using a linear ion trap in “triple play” mode. The coexistence of proteins released by cercariae and host skin proteins from epidermis and basement membrane confirmed that cercarial tunnels in skin were sampled. Among the abundant proteins secreted by cercariae was the cercarial protease that has been implicated in degradation of host proteins, secreted proteins proposed to mediate immune invasion by larvae, and proteins implicated in protection of parasites against oxidative stress. Components of the schistosome surface tegument, previously identified with immune serum, were also released. Both lysis and apoptosis of epidermal cells took place during cercarial invasion of the epidermis. Components of lysed epidermal cells, including desmosome proteins which link cells in the stratum granulosum and stratum spinosum, were identified. While macrophage-derived proteins were present, no mast cell or lymphocyte cytokines were identified. There were, however, abundant immunoglobulins, complement factors, and serine protease inhibitors in skin. Control skin samples incubated with water for the same period as experimental samples ensured that invasion-related proteins and host protein fragments were not due to nonspecific degeneration of the skin samples.

**Conclusions:**

This analysis identified secreted proteins from invasive larvae that are released during invasion of human skin. Analysis of specific host proteins in skin invaded by cercariae served to highlight both the histolytic events facilitating cercarial invasion, and the host defenses that attempt to arrest or retard invasion. Proteins abundant in psoriatic skin or UV and heat-stressed skin were not abundant in skin invaded by cercariae, suggesting that results did not reflect general stress in the surgically removed skin specimen. Abundant immunoglobulins, complement factors, and serine protease inhibitors in skin form a biochemical barrier that complements the structural barrier of the epidermis, basement membrane, and dermis. The fragmentation of some of these host proteins suggests that breaching of host defenses by cercariae includes specific degradation of immunoglobulins and complement, and either degradation of, or overwhelming the host protease inhibitor repertoire.

## Author Summary

Understanding the molecular and biochemical mechanisms by which parasites invade their host is key to rational vaccine and drug development. The proteins released by larvae of a parasitic blood fluke as they invade human skin were identified using high-resolution protein identification equipment and software. In addition, the proteins in human skin that serve as mechanical or biochemical barriers to parasite invasion were also identified. Among the proteins released by the parasite larvae were enzymes that degrade host tissue and immune defense molecules and other factors that protect the larvae from host defense mechanisms. Analysis of specific host proteins in skin invaded by cercariae served to highlight both the tissue degradation that facilitates cercarial invasion, and the host defenses that attempt to arrest or retard invasion. Proteins abundant in other skin diseases or UV and heat-stressed skin were not abundant in skin invaded by cercariae, suggesting that results did not reflect general stress in the surgically removed skin specimen. Abundant immune response proteins in human skin form a biochemical barrier that complements the structural barrier of the epidermis, basement membrane, and dermis. The fragmentation of some of these host proteins suggests that breaching of host defenses by cercariae includes specific degradation of immune response proteins and overwhelming the host protease inhibitor repertoire.

## Introduction

Human skin is a highly evolved barrier to environmental pathogens [1]. Flattened, keratinized epidermal cells, made adherent by a lipid-rich intercellular milieu, constitute the most superficial barrier called the stratum corneum. This cornified layer of cells is derived from keratinized cells in the underlying stratum granulosum. Beneath this layer, large glycogen-rich epidermal cells form a cellular matrix called the stratum spinosum. Cells in the stratum spinosum are locked together by multiple dense intercellular bridges formed by protein complexes called desmosomes. The epidermis is also anchored to a basement membrane that separates it from the dermis [1].

In the absence of mechanical or thermal trauma, the skin is a formidable barrier. However, there are instances where pathogens gain access to the human host by penetrating skin in the absence of trauma. One of the most remarkable examples is the multicellular larva of the schistosome blood fluke called a cercaria (ae). These larvae, 150 µ long×70 µ wide, are composed of approximately 1000 cells that include organ-like clusters adapted to host finding, locomotion, and invasion [2],[3]. Cercariae are “shed” from an intermediate host snail in aquatic environments. These larvae utilize a variety of environmental cues to find their host, including motion, light-dark contrast, and chemical and thermal gradients [4],[5]. In the case of schistosomes that infect humans, the lipid on the surface of the skin serves not as a barrier but as a signal for invasion [3],[4],[5],[6]. Cercariae may respond to 12 to 14 carbon-free fatty acids in skin lipid as a signal to begin penetration behavior [4]. This behavior consists of rapid motion of the tail followed by release of two sets of acetabular “glands”, which contain an adhesive mucin-like substance, proteolytic enzyme activity, and proteins implicated in immune evasion [6],[7],[8].

Upon interaction with human skin in fresh water, cercariae begin to invade by entering the hydrated superficial cornified layer. Invasion through the epidermis is clearly facilitated by acantholysis, which is characterized by degradation of desmosome cross-links that tether epidermal cells together [9],[10]. Following degradation of the epidermal basement membrane, cercariae transit through the dermal extracellular matrix to enter small venules or lymphatics in the superficial dermis.

Two previous proteomic studies identified the major proteins secreted when cercariae were artificially induced to invasive behavior *in vitro*. Most proteins are secreted from vesicles of the major secretory cells of the larva called acetabular glands, [11],[12]. In contrast, uninduced free swimming cercaria release relatively small number of non-acetabular gland proteins including enolase, GST-28, and actin 2 [12]. The major secreted proteins include a gene family of histolytic serine proteases that likely facilitate degradation of host tissue barriers, and factors that may contribute to immune evasion [8],[13].

Because the previous analyses of cercarial secretion utilized artificial methods to induce invasive behavior, the molecular details of the host-parasite interaction in skin have remained murky. In the present study, we identified which proteins are actually released by cercariae penetrating human skin. The results of this study support previous hypotheses concerning the character of proteolytic activity that cercariae use for invasion, and factors contributing to proposed mechanisms of immune evasion. The results also provide for the first time a glimpse at host-parasite interactions involving host-derived protease inhibitors and complement-mediated responses to invading larvae.

## Materials and Methods

### Maintenance of schistosome lifecycle and collection of cercariae

A Puerto Rican strain of *S. mansoni* was used for all experiments. This isolate was originally obtained from Dr. Fred Lewis of the Biomedical Research Institute, Bethesda, MD, but has been maintained in our laboratory for over 20 years.

*Biomphalaria glabrata* snails are used as intermediate hosts and are maintained in a BSL2 laboratory in accordance with all approved biosafety protocols. *B. glabrata* snails were maintained with a diet of organic lettuce and school chalk as a calcium supplement. All snails were housed in the absence of light to increase yields of cercariae during light induction. Cercariae are obtained using a light induction method described previously [14].

### Invasion of human skin by schistosome larvae

**Figure 1:**
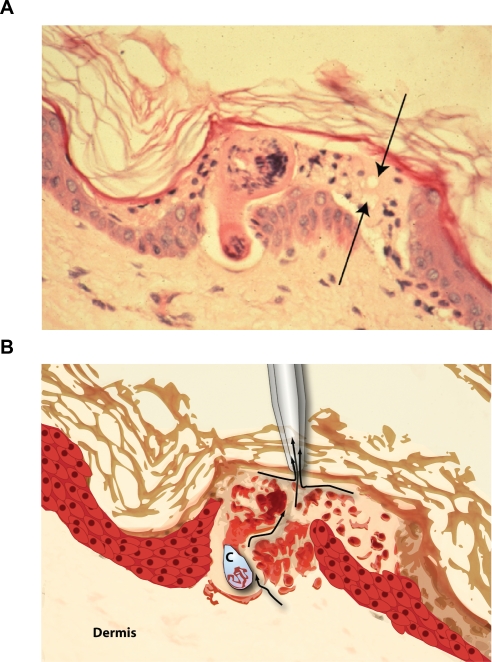
Cercaria and invasive tunnel in epidermis of human skin at 1/2-hour post invasion. A). The parasite larva is just entering the dermis toward the bottom of the figure. Note tunnel (arrows) formed from destruction of epidermal cells by both acantholysis and apoptosis. It is fluid from these tunnels that was targeted for proteome analysis. B) Model of presumed acquisition of fluid from skin invaded by schistosome cercariae. Proteins from the tunnels produced by cercariae (C), as well as lysed epidermal cells and dermal fluid are identified in Tables 1 and 2.

The human skin sample was taken in compliance with protocols approved by the Committee on Human Research at the University of California, San Francisco. Written informed consent was obtained for the operation and use of tissues removed. 3×3 cm human skin samples including epidermis and dermis were obtained from a surgical amputation of the lower extremity in a patient with peripheral vascular disease. The skin was taken from the proximal extremity, and no gross or microscopic histopathology was evident in this region. The samples used for proteomic analysis was taken from skin two hours following surgical removal. Human skin samples were clamped dermal side down to plastic wells (Costar 24 well) containing RPMI 1640 medium at 37°C. Approximately 3000 cercariae, enough to ensure sufficient products were released from the skin, were then applied in 1 ml of water (22°C) into 15-mm plastic cylinders on the exposed skin surface. After 30 or 120 minutes, the fluid was collected by a 1-ml pipette (Gilson pipetman) with suction applied to the skin surface to retrieve fluid from the tunnels produced by cercariae in the epidermis (Figure 1A,B). Any cercariae remaining were removed by centrifugation @ 16 K×G for 10 min. The sample was frozen at −20°C until processed. The experiment was performed at 30 minutes and at 120 minutes post exposure to cercariae.

### Control skin samples

Control skin samples were harvested from the amputation specimen and incubated for the same period of time, dermal side down on 37°C media, as in experimental sets. In addition, skin samples were punctured 10 times with a 27-gauge needle to mimic the tunnels produced by cercariae. This was to rule out that the tunnels themselves served as conduits for degenerating host skin proteins to leach into the experimental samples.

### Epidermal cell apoptosis

Epidermal cell apoptosis was documented microscopically using the TUNEL Assay. Five-micron sections of human skin invaded by cercariae were processed and stained using the Apop Tag Peroxidase In situ Detection Kit (Chemicon, Millipore, Billerica, MA) according to instructions provided by the supplier.

### Isolation and separation of proteins released by cercariae or present in human skin during cercarial invasion

The frozen samples were lyophilized and resuspended in equal volume of 2× Tris glycine SDS sample buffer, and equal volumes were loaded on the gel with an open lane between samples. SDS PAGE was performed on the samples using Tris glycine 4–20% acrylamide gels (Biorad, Hercules, Ca) and See-Blue Plus 2 standards (Invitrogen, Carlsbad, Ca) to calibrate the molecular weight range. The gels were stained in Coomassie blue. This gel is shown in Figure S1.

#### In-gel digestion

The SDS-PAGE gel protein sample lanes were evenly sliced, all slices were reduced with dithiothreitol, alkylated with iodoacetamide, and then subjected to in-gel digestion with side chain-protected porcine trypsin (Promega, Madison, WI) (Detailed protocol: http://ms-facility.ucsf.edu/ingel.html).

#### Fractionation and mass spectrometry

The resulting peptides were subjected to LC/MS/MS analysis. Peptide fractionation was accomplished by reversed phase chromatography using an Ultimate HPLC pump and a Famos autosampler (LC Packings, Dionex, Sunnyvale). Typically ∼1/10^th^ of the digest (1 µl) was injected onto a nanoHPLC column (C18, 75 nm×150 mm), at a flow rate of ∼300 nL/min. Solvent A was 0.1% formic acid in water, solvent B was .01% formic acid in acetonitrile. The column was equilibrated at 5% B and typically a linear gradient up to ∼40% B was developed in 35 min. The samples were analyzed using a linear ion trap (LTQ, Thermo, San Jose) in “triple play” mode: full scan, 10 Da zoom-in on the most abundant ion, MS/MS experiment only on multiply charged precursor ions. Dynamic exclusion was enabled.

#### Data processing and protein identification

LTQ data were processed using Mascot Distiller v.2.1.0.0. and its default parameters recommended for ion trap data.

Database searches were performed using in-house ProteinProspector v. 4.25.2. Searches were performed first on the UniProt.2006.10.21. database implemented with the reversed sequences of all entries. Only *Homo sapiens*, *S. japonicum* and *S. mansoni* sequences were searched (161326/7065562 entries). Search parameters were as follows. Only tryptic peptides were considered, cleavage in front of Pro residues and one missed cleavage were permitted. Carbamidomethylation of Cys residues was considered a fixed modification, while variable modifications included acetylation of protein N-termini, Met oxidation, and pyroglutamate formation from N-terminal Gln residues. Mass accuracy of the precursor ions was within 2 Da. Mass accuracy of the fragment ions was within 0.8 Da.

The acceptance criteria were a minimal peptide score of 15, a minimal protein score of 22, a maximum expectation value of 0.1. Considering the peptides identified from the reversed sequences the false positive rate (FPR) was below 0.5%. (FPR = 2× number of reversed peptides IDd/all the peptides IDd). A second data base search was performed on the samples containing *S mansoni* cercaria, against the Sanger data base (genedb.org/genedb/smansoni) v3.1 (dna_frame_translation: 6; 50292 entries) using the same search parameters as above and the same acceptance criteria. Peptide sequences matched to *Schistosoma* species other than *mansoni* were further BLAST searched against the expressed sequence tags available from The Institute for Genomic Research (TIGR) *S. mansoni* genome project in version 5.0 (www.tigr.org/tdb/tgi/smgi/) [15].

## Results

### Proteins released by invading larvae

Histological sections of human skin at 30 and 120 minutes post-exposure to cercariae showed approximately 90% of invaded larvae in the epidermis, forming tunnels by destruction of epidermal cells (Figure 1A). The remaining 10% of cercariae had already reached the superficial dermis. One-half hour after cercariae initiated invasion of human skin, multiple tryptic peptides from cercarial acetabular gland proteins were identified (Table 1). These included the cercarial elastase (aka cercarial protease) isoforms 2a, 1a and 1b. This is the serine protease implicated in degradation of host tissue barriers [14]. A schistosome serpin (serine protease inhibitor) was also identified and corresponds to that previously reported in cercarial secretions released into media in vitro [11]. Tryptic peptides were identified for schistosome paramyosin; a myosin-like protein implicated in immune evasion, and previously localized by immuno-electron microscopy to the acetabular glands [16],[17]. Heat shock proteins, HSP 86 and HSP 70 had also been identified as major components of acetabular gland secretions in a previous proteomic analysis [12]. SPO-1 (Sm16), an acetabular gland secreted protein implicated in immune evasion [18] was released into human skin.

**Table 1:** Cercarial Proteins with Proposed Biological Function Released During Skin Invasion.

ID a	other Sm ID a ^,^ b	30 min d	2 hrs	Name c
P53442		31	65	Fructose-bisphosphate aldolase
P12546		9	21	Cercarial protease precursor (elastase 1a)
Q8MUV8		5*	13*	Elastase 2a * not overlapping with P12546
Q26552		13**	0	Elastase (elastase 1b) ** not overlapping with P12546 and Q8MUV8
Q6LA98		0	0	Cercarial elastase HP1 no unique peptides for this isoform
Smp_contig038044	Smp_006510	0	1	elastase-like protein
P06198	TC20786	29	0	Paramyosin
Q05870	TC20786	3	0	Paramyosin, SCHJA only unique sequences
P35661		7	10	Glutathione S-transferase class-mu 26 kDa isozyme
P09792		3	10	Glutathione S-transferase class-mu 28 kDa isozyme
Q86LC0		0	2	Glutathione S-transferase omega
P32070		6	10	Antigen Sm21.7
P08418		6*	1	Heat shock 70 kDa protein homolog *3 sequences shared with human
Q8T9N5		0	10	Thioredoxin
Smp_contig00147	Smp_003300	1	2	Serpin C
O77234		3	3	Stathmin-like protein
P91804		4	1	Tegumental antigen Sm20.8
Q9Y0D3		0	1	Thioredoxin peroxidase
Q26557		1	0	Gynecophoral canal protein
P27730		1	0	Calpain
Smp_contig025163	Smp_089460.2	1	0	Calpain III
P15845		1	0	20 kDa calcium-binding protein
Smp_contig000595	Smp_123090	0	1	venom allergen-like protein 4

Number of peptides IDd, all replicates counted.

Additional proteins of cytosokic orign, low abundance, or unknown function can be viewed as: Table S1.

Mass spec data and peptides for Table 1 can be viewed as: Table S2, 30 min cercaria, and Table S3, 2 hr cercaria.

Mass spec data and peptides for Table S1 can be viewed as: Table S4, 30 min cercaria, and Table S5, 2 hr cercaria.

a mass spectra matched Uniprot database protein accession numbers. Multiple accession numbers indicate that an ID in the SANGER and/or TIGR database encompassed all the peptides associated with the individual IDs.

b mass spectra matched Sanger contigs numbers; these represent proteins with peptides found in addition to those identified in the Uniprot database.

c name assigned by Uniprot. Brackets indicate names from Sanger BLAST search.

d Proteins have been sorted based on the 30 minute skin exposure, from highest to lowest. In some cases, proteins have been grouped by name or function (ie elastases, GSTs).

Two schistosome-specific proteins, Sm20.8 and Sm21.7, previously reported to be components of the schistosome surface or subjacent tegument [19],[20], and two glutathione-S-transferases, GST 26 and GST 28, were released from the parasite. Finally, a group of schistosome glycolytic enzymes and kinases, known to be abundant in the cytoplasm of larval gland cells were identified. These are presumbably released as holosecretion from the acetabular cells [11],[12] and included glyceraldehyde-3-phosphate-dehydrogenase, glycerol phosphorylase, triose phosphate isomerase, fructose-1-6 adolase, cytosolic ATP-guanidine kinase, and enolase.

At two hours post invasion, many larval proteins increased in abundance including GST 28, GST 26, the elastase isoforms, and the glycolytic enzymes. New proteins were detected at 120 minutes, notably schistosome-derived thioredoxin, thioredoxin peroxidase and superoxide dismutase [21]. A venom allergen-like protein was secreted at 120 minutes. This protein had been identified in larval secretions released into media during a 3 hour in vitro collection [11]. The venom allergen-like protein was proposed to have immuno-modulating activity by its structural similarity to chemokines [11],[22]. The calcium-binding proteins, calponin and CBP, which are secreted from the acetabular glands, were also detected [12],[23]. In contrast, paramyosin secretion decreased dramatically at 120 minutes.

### Proteins detected in control human skin samples

To ensure that fragmentation of host proteins produced by cercarial invasion, or accumulation of host proteins due to interaction with secreted parasite proteins were selectively identified, samples of human skin incubated in an identical manner, but not exposed to cercariae, were analyzed as controls. To control for any degeneration that might have occurred within the timeframe from skin harvesting to the collection of protein samples, human skin samples harvested from the same surgical specimen were incubated dermal side down in media for the same length of time as experimental samples exposed to invading cercariae. The same amount of water used to apply cercariae was applied to the control skin surface. Control skin samples were also punctured 10 times with a 27-gauge needle to mimic the tunnels produced by cercariae. Therefore, any proteins that might have leached into these tunnels from adjacent skin would be noted. In Table 2, a comparison of experimental and control proteomes is indicated.

**Table 2:** Quantitative Comparison of Human Proteins Detected.

Number of peptides identified						
Acc#	exp.30min*	exp.2h	C30min**	C2h	C2hpunct***	Name
Keratins						
various	487	866	360	815	869	type 2 keratins
various	302	406	194	460	596	type 1 keratins
Q5DT20	0	8	0	7	44	Hornerin
**Protease inhibitors (serum and intra cellular)**						
P01023	15	52	117	161	102	Alpha-2-macroglobulin precursor
P01009	24	39	56	83	57	Alpha-1-antitrypsin precursor
P05155	2	0	15	15	7	Plasma protease C1 inhibitor precursor
P01011	5	4	8	6	4	Alpha-1-antichymotrypsin precursor
P01008	3	4	4	1	5	Antithrombin-III precursor
P61769	0	0	0	1	0	Beta-2-microglobulin precursor
P02760	0	0	0	2	1	AMBP protein precursor [Contains: Alpha-1-microglobulin]
Q3SYB4	0	2	0	0	0	SERPINB12 protein
P35237	0	1	0	0	0	Serpin B6
P01042	0	1	0	1	1	Kininogen-1 precursor
P01040	0	0	0	1	0	Cystatin-A
O75635	0	1	0	0	0	Serpin B7
P05543	0	0	1	0	0	Thyroxine-binding globulin precursor
**Immunoglobulins and major serum proteins**						
P02768	353	483	385	546	514	Serum albumin precursor
P02766	7	8	12	23	17	Transthyretin precursor
P43652	0	0	1	1	0	Afamin precursor
Q9UL85	0	3	0	5	2	Myosin-reactive immunoglobulin kappa chain variable region
Q6GMY2	0	3	0	10	4	IGHM protein
P01620	2	6	0	10	5	Ig kappa chain V-III region SIE
P01876	3	11	14	20	14	Ig alpha-1 chain C region
Q6IN78	19	105	30	63	58	IGHG1 protein also Q6PJ95
Q6GMV9	16	45	21	58	33	IGKC protein also Q6PIH6
P68871	49	69	41	90	82	Hemoglobin subunit beta
P69905	25	45	30	63	60	Hemoglobin subunit alpha
P02787	16	73	48	74	51	Serotransferrin precursor
P06727	0	1	7	4	6	Apolipoprotein A-IV precursor
P05090	0	6	4	6	5	Apolipoprotein D precursor
P02647	11	15	25	46	20	Apolipoprotein A-I precursor
P02649	1	0	10	7	4	Apolipoprotein E precursor
P00450	1	36	21	52	31	Ceruloplasmin precursor
P00738	3	19	23	26	25	Haptoglobin precursor [ Haptoglobin alpha chain; Haptoglobin beta chain]
P02679	2	17	8	24	11	Fibrinogen gamma chain precursor
P02671	0	4	6	12	1	Fibrinogen alpha chain precursor [Contains: Fibrinopeptide A]
P02675	0	6	4	10	8	Fibrinogen beta chain precursor [Contains: Fibrinopeptide B]
Q6GTG1	6	11	18	8	7	Group-specific component
P02753	0	2	3	3	1	Plasma retinol-binding protein precursor
P15090	2	1	2	2	1	Fatty acid-binding protein, adipocyte
P31151	2	0	0	0	0	Protein S100-A7
P02763	9	12	10	16	16	Alpha-1-acid glycoprotein 1 precursor overlapping with Q5T539, Q5U067
P19652	2	5	2	5	5	Alpha-1-acid glycoprotein 2 precursor overlapping P02763,unique listed
P25311	2	8	5	5	3	Zinc-alpha-2-glycoprotein precursor
P02790	4	4	2	6	6	Hemopexin precursor
P04217	0	6	5	6	4	Alpha-1B-glycoprotein precursor
P02765	0	0	1	5	4	Alpha-2-HS-glycoprotein precursor
P04196	0	1	0	0	0	Histidine-rich glycoprotein precursor
P02743	0	1	3	5	4	Serum amyloid P-component precursor
P02735	0	0	1	2	0	Serum amyloid A protein precursor
P35542	0	0	1	1	1	Serum amyloid A-4 protein precursor
Q49AQ8	0	0	0	0	2	SELENBP1 protein
P02144	0	2	4	9	2	Myoglobin
**Complement associated proteins**						
P01024	5	10	23	39	13	Complement C3 precursor
P00751	0	0	3	6	6	Complement factor B precursor
P02748	0	1	0	0	0	Complement component C9 precursor
P07360	0	0	0	1	0	Complement component C8 gamma chain precursor
P0C0L4	0	0	1	7	0	Complement C4-A precursor
Q4LE82	0	1(2)	0	0(7)	0	C4A variant protein 1 unique sequence, all others shared with P0C0L4
P10909	1	0	4	2	3	Clusterin precursor
**Epidermal cell or Desmosome-associated proteins**						
P60709	34	53	14	32	26	Actin, cytoplasmic 1
P06396	0	9	1	2	4	Gelsolin precursor
Q01995	0	0	1	2	2	Transgelin
P12273	0	0	1	0	0	Prolactin-inducible protein precursor
Q02413	5	3	3	8	5	Desmoglein-1 precursor
P08670	0	0	4	7	1	Vimentin
Q5D862	2	2	0	0	1	Ifapsoriasin
Q15151	0	0	0	1	2	Plakoglobin
P15924	0	0	0	0	2	Desmoplakin also P14923
Q13748	5	0	0	0	0	Tubulin alpha-3C/D chain
Q13509	3	0	0	0	0	Tubulin beta-3 chain
P68371	5	0	0	0	0	Tubulin beta-2C chain
P62158	0	0	1	1	0	Calmodulin
**Basement membrane or matrix proteins**						
P51884	2	9	20	15	11	Lumican precursor
P23142	0	0	1	4	3	Fibulin-1 precursor
O95617	0	0	0	1	0	Fibronectin
Monokines						
P07333	0	0	0	1	0	Macrophage colony-stimulating factor 1 receptor precursor
P14174	0	0	0	1	0	Macrophage migration inhibitory factor
**Cytosolic proteins**						
P08107	1	0	0	0	0	Heat shock 70 kDa protein 1
O75322	0	1	0	0	0	Hsp89-alpha-delta-N
P11142	1(4)	0	0	0	0	Heat shock cognate 71 kDa protein 1 unique sequence for human
P02792	0	0	1	2	1	Ferritin light chain
Q86SE5	0	1	0	2	1	RNA-binding Raly-like protein
O60814	0	0	0	0	2	Histone H2B type 1-K
P16104	1	0	0	0	0	Histone H2A.x
P62805	4	0	0	0	2	Histone H4
Q14865	0	0	0	0	1	AT-rich interactive domain-containing protein 5B
P32119	3	1	8	6	4	Peroxiredoxin-2
P30041	0	0	0	4	1	Peroxiredoxin-6
Q06830	0	0	2	0	0	Peroxiredoxin-1
P00441	0	0	0	2	0	Superoxide dismutase [Cu-Zn]
P30043	0	1	1	1	0	Flavin reductase
P62937	2	1	6	2	1	Peptidyl-prolyl cis-trans isomerase A
P00915	10	19	10	14	14	Carbonic anhydrase 1
P00918	1	0	3	4	0	Carbonic anhydrase 2
P07451	0	2	1	1	0	Carbonic anhydrase 3
P06744	0	2	0	0	0	Glucose-6-phosphate isomerase
P04406	0	1	1	1	0	Glyceraldehyde-3-phosphate dehydrogenase
P06733	0	1	0	0	0	Alpha-enolase
P60174	0	1(2)	0	1(2)	0	Triosephosphate isomerase 1 unique human sequence
P25705	4	0	0	0	0	ATP synthase subunit alpha, mitochondrial precursor
P40925	0	0	0	1	0	Malate dehydrogenase, cytoplasmic
P00338	0	0	0	1	0	L-lactate dehydrogenase A chain
P09668	0	1	0	0	0	Cathepsin H precursor
P31944	0	1	1	1	0	Caspase-14 precursor
P07339	1	3	1	1	0	Cathepsin D precursor
P06732	0	0	1	1	0	Creatine kinase M-type
P00558	0	9	0	0	0	Phosphoglycerate kinase 1 some overlapping with parasite protein
P63104	0	0	1	0	0	14-3-3 protein zeta/delta
P61626	0	0	1	0	0	Lysozyme C precursor
Q6B0J6	0	0	1	0	0	Paraoxonase 1

All replicate identifications above the acceptance threshold were counted.

Proteins in boxes are related.

Mass spec data and peptides for Table 2 can be viewed as: Table S6, 30 min cercarial exposure, human peptides; Table S7, 2 hr cercarial exposure, human peptides; Table S8, 30 min control human peptides; Table S9, 2 hr control human peptides; and Table S10, 2 hr puncture human peptides.

*: exp: cercaria on skin.

**: C: water on skin.

***: : water on punctured skin.

Comparison of results from control human skin samples to proteomes derived from living human skin also helped to confirm that the fluid retrieved for proteomic analysis came from skin that had not undergone significant degeneration. Several proteins identified in Table 2 were previously identified in a proteomic analysis of suction blister fluid [24]. These include amyloid protein, clusterin, colony stimulating factor, cystatin, and haptoglobin-related protein. Suction blister fluid reflects the protein composition present in the interstitial fluid that bathes epidermal cells [24]. While derived from serum, it is filtered through the epidermal basement membrane. Proteins such as host cystatin and aspartyl protease precursor, may also derive from sweat glands in the skin [25]. There were many serum proteins, including immunoglobulins, apolipoprotein, haptoglobin, alpha-2 macroglobulin, complement C4, C3, and C2b (the complement protease fragment), and fibrinogens (alpha, beta, and gamma). Some of these proteins are found in suction blister fluid [22], but many are more reflective of serum proteins that bathe the dermis and likely entered the samples because of the 27-guage-needle puncture of the epidermal-dermal basement membrane. In either case, their presence suggests that in addition to the structural barriers presented by epidermis, basement membrane, and dermis, host defense factors, such as complement components, immunoglobulins and protease inhibitors, are already present in skin. As discussed below, alterations in the size or abundance of these host defense molecules were noted in skin invaded by cercariae.

### Human skin and serum proteins associated with or altered by invading larvae

**Figure 2:**
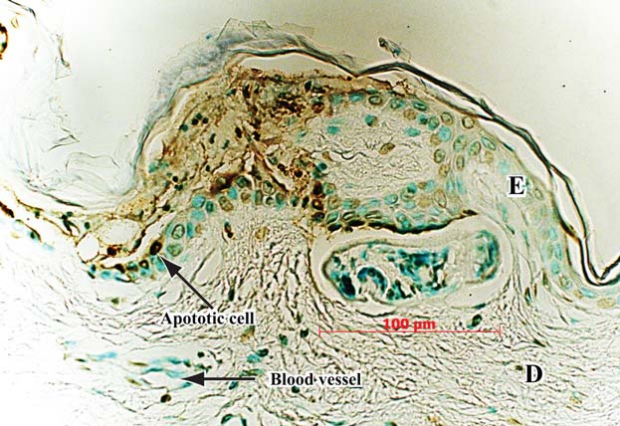
Tunel assay demonstrating apoptosis of epidermal cells (punctate brown immunoperoxidase staining on left). A cercaria is immediately above 100-micron bar. It is now in the dermis (D), but had invaded epidermis from left to right. Internal control is absence of apoptotic signal in intact epidermis on right (E).

Cercarial invasion induced epidermal cell apoptosis as documented by a positive TUNEL assay (Figure 2), and acantholysis as visualized microscopically (Figure 1A). By comparison to control skin samples, three host skin proteins were clearly degraded by invading larvae (Table 2). The first was gelsolin, an actin-binding and dissociation protein found both in cell cytoplasm and in plasma [26]. The human gelsolin peptides were only found at 40 kDa in the invaded skin samples, but at 80 kDa in the control skin. The second was C1 inhibitor, a serum-derived inhibitor of complement factors, C1r and C1s [27]. The third was desmoglein, a desmosome-related protein [28] which appeared as a smaller fragment in skin invaded by cercariae, but no unique tryptic peptide was present compared to mechanically disrupted controls. The lysis of epidermal cells in the wake of invading cercariae released numerous desmosome – related proteins and epidermal cell cytosolic proteins (Table 2) including keratin isoforms.

There was a striking accumulation of host serine protease inhibitors (serpins) in both control and experimental skin samples. These included alpha-2-macroglobulin, alpha-1-antitrypsin, anti-chymotrypsin, and antithrombin. In the samples invaded by cercariae, the number of alpha-1-antitrypsin, and alpha-2-macroglobulin peptides was greatly reduced. C1 inhibitor was greatly reduced in both number and size. The specific fragmentation of proteins seen in the presence of cercariae, is distinctly different from the protein repertoire in control skin, and complement factors were not seen in suction blister fluid [24]. Notably there was an absence of the human skin stress- protein HSP 27 which is abundantly induced in skin with thermal or UV-stress [29]. In a proteome analysis of psoriatic skin, proteins that increase more than twofold included cytokeratins, HSP 27, and 14-3-3 [30]. Cytokeratin release correlated with epidermal cell lysis in psoriasis and was similarly seen with cercarial invasion.

## Discussion

This study identified proteins in human skin that are relevant to the biology of the host-parasite interplay occurring during initial infection of the human host by *S. mansoni* cercariae. The results validate, clarify, or extend analyses of invasive behavior of cercariae observed *in vitro*, and present a molecular view of invasion of skin by a human parasite in as “biological” a situation as is ethically possible. That said there are likely other relevant protein factors that might not be present due to ethical limitations. First, while this is recently harvested human skin, it is no longer perfused from the vascular system. Components of the bloodstream that might reach the site of invasion by chemotaxis may not be present. The resolution of mass spectrometry following tryptic digest may also not identify peptide fragments generated from host proteins that are targets for cercarial protease, or other protease activity. Nevertheless, the rich dataset of both parasite and host skin proteins, discussed below, provides the most complete picture to date of the pathobiology of invasion. The experiment was performed twice (only one experiment is reported here), and control skin samples incubated in water in an identical manner ensured that host proteins identified as bound or fragmented by larval invasion were not present due to nonspecific skin degeneration (Table 2). Indeed the abundant proteins induced in UV-stressed or heat-stressed skin in previous studies [29] were either not abundant (HSP 70) or even present (HSP 27) in these skin samples. The coexistence of proteins released by cercariae and host skin proteins from epidermis and basement membrane confirmed that cercarial tunnels in skin were sampled (Figure 1A,B).

Invasion of human skin by schistosome blood fluke larvae is a remarkable biological process in which a multicellular, 0.1 mm long parasite larva breaches the epidermis, basement membrane, and dermal barriers of the skin [3]. This occurs without disruption by the bite of an insect vector or trauma. Previous studies have implicated a small gene family of closely related serine proteases, called cercarial elastase, in facilitating this invasion in one species of schistosome, *S. mansoni* [13],[14]. The proteins released during invasion are different than those found in media from free swimming larvae [12]. Cercarial elastase was identified as a major component of acetabular gland secretions by proteomic analyses of artificially activated cercariae [11],[12]. This present analysis of proteins detected during cercarial invasion of recently harvested human skin confirms that this protease is a major secretory product released during skin invasion. Most of the peptides detected as cercarial elastase were in the range of 26 to 37 kDa on a one-dimensional SDS-PAGE. This is consistent with the intact catalytic domain of the enzyme. However, peptides were also detected in the 15 to 19-kDa range, consistent with a known autocatalytic product of cercarial elastase turnover identified in biochemical studies as 17 kDa [31]. Cercarial elastase peptides were also found at 64 kDa in early invasion, along with alpha-1-antitrypsin peptides, suggesting the formation of a complex.

Infection with the human parasite used in these studies, *Schistosoma.mansoni*, only causes cercarial dermatitis after multiple exposures in an experimental vaccine model with radiation-attenuated cercariae. However, cercarial dermatitis commonly occurs with invasion by cercariae from bird or small mammal schistosomes like those of the genus Trichobilharzia. One would not expect to detect abundant inflammatory mediators in the skin used here which recapitulates naive exposure of human skin to *S. mansoni* cercariae [32]. During invasion of mammalian skin, larvae of *S. mansoni* elicit minimal immune responses [33],[34]. This is thought to be due to the parasite's physical and biochemical adaptations in a new environment including loss of the cercarial tail and glycocalyx, the formation of a unique surface bilayer, and the release of acetabular proteins with immunoregulatory properties. Among these latter proteins are the cercarial elastase itself, which may degrade host immunoglobulins [35], as well as SPO-1, T-cell stimulating antigen (11 kD) and paramyosin. SPO-1 is hypothesized to be an anti-inflammatory protein secreted by schistosomula during skin invasion [18]. Paramyosin is itself very immunogenic and has been proposed to protect the parasite from immune attack by ”decoy” binding proteins of the complement pathway [17].

Other key proteins secreted by cercariae into human skin included a calcium-binding protein previously identified as a component of acetabular gland secretions [12],[23]. The so-called “pre-acetabular glands” are known to contain high concentrations of calcium, the exact function of which unknown, although it was proposed that a high calcium concentrations might serve as post-translational regulation of protease activity [36].

At the later (120 min) stage of invasion, cercariae released enzymes implicated in protection against oxidative stress, including GST-26, thioredoxin peroxidase and superoxide dismutase [21],[37],[38]. At that time point, microscopy revealed that more cercariae had reached the lower border of the epidermis or were entering the superficial dermis and approaching the vascular compartment. Here they may encounter host defense factors from dermal inflammatory cells or serum that include reactive oxygen species.

In the two-hour sample, there were also proteins released that were known to be present on the tegument of schistosomula, the stage that first enters the bloodstream. These include Sm20.8, Sm21.7 and calpain. These proteins were presumably either shed by invading schistosomula, or were released from dying or damaged worms. In either case the detection of these parasite proteins explains why they were recognized by the immune system in animal models of infection [19],[20],[39].

Finally, a group of known schistosome cytosolic enzymes and proteins, including several abundant glycolytic enzymes, were detected. While the presence of these molecules may reflect proteins released by damage to the tegumental cell layer during invasion, histological analysis of cercariae and schistosomula at 1/2 hour or 2 hours in this study showed only intact organisms with no evidence of cellular degeneration or tegument blebbing. More likely these proteins represent cytosolic components of the acetabular “glands” or secretions of the excretory apparatus following cercarial tail loss. The acetabular “glands” are in fact cells whose processes extend to the anterior end of the organism, and release cellular holosecretion [12]. Therefore, it is not surprising that cytosolic components are released into the host. Indeed, these and the tegumental proteins discovered in host tissue were first identified as immunogenic proteins during host infection, suggesting they were “seen” by the host immune response [19],[20],[40]. Several were pursued as potential vaccine components. A previous proteomic study of cercarial secretions isolated *in vitro* confirmed the presence of these enzymes in acetabular gland cells [12].

High-molecular weight host cytokeratins and other epidermal cytosolic proteins were abundant and likely represent proteins released from epidermal cells lysed in the wake of invading cercariae (Figure 1A,B). Cytokeratins were also identified in a proteome analysis of psoriatic skin in which epidermal cell lysis is a hallmark [30]. Caspase 14 was detected in skin invaded by cercariae, suggesting that not only was there epidermal cell lysis, but also that apoptotic pathways were induced during cercarial invasion. This was confirmed by a TUNEL assay (Figure 2). The TUNEL assay was only positive in skin invaded by cercariae and not in adjacent non-invaded skin or in controls. Apoptosis may represent a keratinocyte response to degradation of desmosomes, or to other external signals of tissue disruption. Gelsolin, an 81 kDa native protein, has a 41 kDa fragment associated with caspase 3 degradation during apoptosis [41]. This fragment, determined by relative kDa of the gel slice, appears in the 30 and 120-minute cercaria-exposed skin and not in control skin. An initial skin experiment with cercariae, found 9 40 kDa-fragment peptides in the 30 minute cercarial exposed skin migrating at ∼40 kDa (data not shown). Previous work had identified apoptotic cells in naïve mouse skin exposed to cercariae, as well as increases in caspase-3, but the earliest time point sampled in that study was 8 hours post-exposure [42].

Aside from identifying proteins released by cercariae during skin invasion, this analysis identified abundant host blood serum proteins that perfuse through skin and likely contribute to its biological barrier. Numerous peptides were identified for the protease inhibitors α-1 antitrypsin, α-1 anti-chymotrypsin, and antithrombin, as well as other major serum proteins (Table 2). The detection of three basement membrane proteins in skin suggests a damaged or “leaky” basement membrane through which serum proteins that bathe the dermis might leach into the epidermis. Other major host defense proteins found in skin were immunoglobulins and complement. The identification of multiple peptides for complement factor C3 is notable in that several investigators have suggested that complement-mediated lysis is a key defense element of the host to prevent schistosome infection [43],[44]. A proteomic analysis of adult schistosomes also had identified host-derived immunoglobulin and complement on the surface [45]. During initial invasion, cercariae must avoid complement-mediated damage by releasing a surface glycocalyx [46]. Components of the alternative complement pathway were abundant in the control skin samples. This suggests that complement perfuses from the vascular compartment into the dermis to serve as a biological barrier to pathogen invasion. It is therefore notable that in skin where schistosome cercariae had invaded, the relative abundance of complement-derived peptides was significantly reduced. This may either indicate an activation of the C3 pathway in response to invading cercariae, or degradation of C3 components by cercarial proteases as a mechanism of immune evasion. While C3, clusterin, the complement lysis inhibitor, C1 inhibitor, and factor B were all present in skin, peptides representing these complement components were all reduced 5–15 fold in skin in which cercariae invaded. Only two macrophage products were identified in control skin, and macrophage colony stimulating factor (MCSF-1) and macrophage inhibitory factor (MIF) were lost in skin invaded by cercariae. A likely source of these proteins is the dendritic cells of the epidermis that may be lysed during cercarial invasion. Dendritic cells share a monocyte linage with macrophages and are directly in the path of invading cercariae (Figure 1A).

Relevant to other proposed mechanisms of larval invasion was the fact that although 30 peptides could be identified for C1 inhibitor in matched control skin, but only two peptides were identified in skin in which cercariae invaded. C1 inhibitor may therefore represent a major target for cercarial proteases. Loss of C1 inhibitor results in increased vascular permeability which might facilitate entry of the migrating schistosomulum into the vascular compartment [27].

Finally, it was noteworthy that α-1-antitrypsin and elastase were present in higher molecular weight forms in the same gel slice, consistent with a complex between this inhibitor and the cercarial elastase. *In vitro* studies had shown that α-1-antitrypsin was indeed a potent cercarial elastase inhibitor [31]. In contrast, not only C1 inhibitor, but antithrombin and α-2 macroglobulin were present as lower molecular weight fragments in cercarial-invaded versus control skin.

## Supporting Information

Figure S1 4–20% SDS PAGE gel, stained briefly with Coomasie Blue. Samples are proteins from skin exosed for 30 minutes or 2 hours, to water alone (WATER), 27-guage hypodermicd needle and water (PUNCT) or *S. mansoni* cercaria in ater (CERC). The molecular weight stards are indicated (MW STD). The sample lanes were cut into uniform 1 mm slices from top of the gel, including the stack, to the bottom of the gel.
(0.45 MB PDF)

Table S1 Additional schistosome proteins of cytosolic origin, low abundance, or unknown function.
(0.03 MB XLS)

Table S2 Mass spec data and unique sequences of proteins from Table 1, 30 minute exposure.
(0.05 MB XLS)

Table S3 Mass spec data and unique sequences of proteins from Table 1, 2 hour exposure.
(0.05 MB XLS)

Table S4 Mass spec data and unique sequences of proteins from Table S1, 30 minute cercarial peptides.
(0.08 MB XLS)

Table S5 Mass spec data and unique sequences of proteins from Table S1, 2 hour cercarial peptides.
(0.07 MB XLS)

Table S6 Mass spec data and unique sequences of proteins from Table 2, 30 minute exposure.
(0.07 MB XLS)

Table S7 Mass spec data and unique sequences of proteins from Table 2, 2 hour exposure.
(0.12 MB XLS)

Table S8 Mass spec data and unique sequences of proteins from Table 2, 30 minute control.
(0.10 MB XLS)

Table S9 Mass spec data and unique sequences of proteins from Table 2, 2 hour control.
(0.14 MB XLS)

Table S10 Human proteins identified in the punctured 2 hr skin control listed in order of Table 2.
(0.12 MB XLS)
