# Supplementary figures and images for: Proteomic Analysis of Skin Invasion by Blood Fluke Larvae
Source: PLoS Negl Trop Dis. 2008 Jul 16;2(7):e262. doi: 10.1371/journal.pntd.0000262 (PMC2467291; doi:10.1371/journal.pntd.0000262)

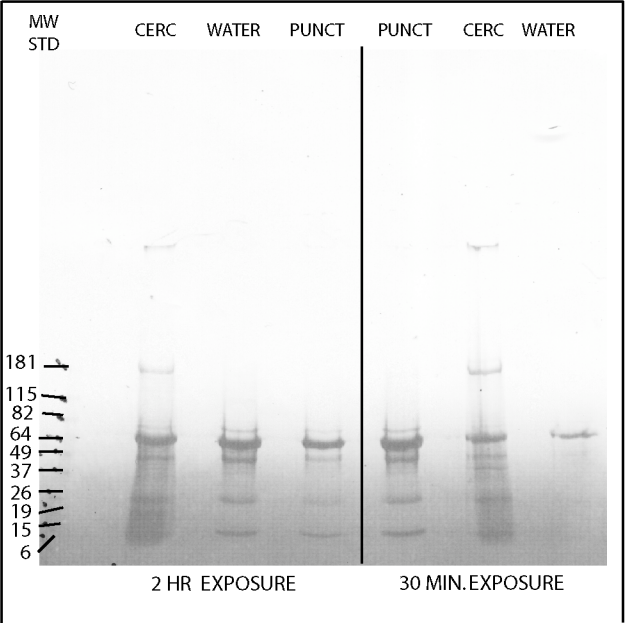

Supplement: Figure S1 — 4–20% SDS PAGE gel, stained briefly with Coomasie Blue. Samples are proteins from skin exosed for 30 minutes or 2 hours, to water alone (WATER), 27-guage hypodermicd needle and water (PUNCT) or S. mansoni cercaria in ater (CERC). The molecular weight stards are indicated (MW STD). The sample lanes were cut into uniform 1 mm slices from top of the gel, including the stack, to the bottom of the gel. (0.45 MB PDF) [file pntd.0000262.s001.pdf]
